# Personalized compression therapeutic textiles: digital design, development, and biomechanical evaluation

**DOI:** 10.3389/fbioe.2024.1405576

**Published:** 2024-06-26

**Authors:** Yu Shi, Rong Liu, Chongyang Ye

**Affiliations:** ^1^ School of Fashion and Textiles, The Hong Kong Polytechnic University, Hong Kong, Hong Kong SAR, China; ^2^ Laboratory for Artificial Intelligence in Design, Hong Kong, Hong Kong SAR, China

**Keywords:** therapeutic biomaterials, biofabrication, compression supply, biomechanical modeling, performance evaluation

## Abstract

Physical-based external compression medical modalities could provide sustainable interfacial pressure dosages for daily healthcare prophylaxis and clinic treatment of chronic venous disease (CVD). However, conventional ready-made compression therapeutic textiles (CTs) with improper morphologies and ill-fitting of pressure exertions frequently limit patient compliance in practical application. Therefore, the present study fabricated the personalized CTs for various subjects through the proposed comprehensive manufacturing system. The individual geometric dimensions and morphologic profiles of lower extremities were characterized according to three-dimensional (3D) body scanning and reverse engineering technologies. Through body anthropometric analysis and pressure optimization, the knitting yarn and machinery variables were determined as the digital design strategies for 3D seamless fabrication of CTs. Next, to visually simulate the generated pressure mappings of developed CTs, the subject-specific 3D finite element (FE) CT-leg modelings with high accuracy and acceptability (pressure prediction error ratio: 11.00% ± 7.78%) were established based on the constructed lower limb models and determined tissue stiffness. Moreover, through the actual *in vivo* trials, the prepared customized CTs efficiently (Sig. <0.05; *ρ* = 0.97) distributed the expected pressure requirements referring to the prescribed compression magnitudes (pressure error ratio: 10.08% ± 7.75%). Furthermore, the movement abilities and comfortable perceptions were evaluated subjectively for the ergonomic wearing comfort (EWC) assessments. Thus, this study promotes the precise pressure management and clinical efficacy for targeted users and leads an operable development approach for related medical biomaterials in compression therapy.

## Introduction

Chronic venous disease (CVD) in biologically lower extremity caused by the valvular incompetence of venous system has been considered as the clinical spectrum ranging from asymptomatic symptoms to venous ulceration (Guo et al., 2019; Kumar et al., 2022). Recently, the prevalence of CVD globally increased from 7.5% to 73% varied by gender, aging, geographic area, and occupational factors (Saliba et al., 2020; Aslam et al., 2022; Gujja et al., 2022; Stücker and Rabe, 2022). Compression therapeutic textiles (CTs)—as the traditional intervention modalities—are extensively applied for the medical treatment and prevention of chronic leg ulcers in physical-based compression therapy (Stücker et al., 2020; Mestre et al., 2022; Xiang et al., 2022). By varying material stiffness, gradient external compressions distributed by elastic knitted CTs could reduce venous wall distension and promote venous hemodynamics from the distal to the proximal regions of lower extremities (Orhurhu et al., 2021; Raffetto et al., 2021). Therefore, CTs lead to beneficial therapeutic functions and support an available solution for CVD clinic treatment and daily healthcare prophylaxis.

In compression management, fabric geometric circumferential dimensions are the size selection criteria of CTs. The applied compression levels—standardized by the measured interfacial pressure dosages at the ankle (B) point—are determined through the medical diagnosis according to a patient’s clinical presentation (Nørregaard et al., 2014). Moreover, the ergonomic wearing comforts (EWCs)—relating to the physical and psychological explorations—primarily impact user compliance with CTs (Nørregaard et al., 2014). The relationships between the body shape, movement freedom, and comfortable perception are crucial for CT EWCs (Jin et al., 2015; Venkatraman et al., 2015). Nevertheless, for existing commercial CTs—due to the regional morphological diversity and measurement discrepancy—the recommended material morphologies and pressure prescriptions from various regions or countries frequently led to the wrong selection and inappropriate applications for different patient groups (Laxa et al., 2016; Jindal et al., 2020). The ill-fitting of CTs commonly increased the risks of threading difficulties, wrinkle, slippage, and discomfort in wearing perceptions (Winslow and Brosz, 2008; Wade et al., 2017), further preventing the compromised therapeutic treatment and exacerbated the unexpected tourniquet effects (i.e., skin break, ulcers, blister, and mobility difficulty) (Nelson and Bell, 2014; Buset et al., 2021; Schupke et al., 2014). The major causes of the poor adherence included the intrinsic disadvantages of the CTs, knowledge gaps in the treatment or management, few recommendations from medical staff, and other sociopsychological factors (Naci et al., 2020). Especially, the established morphology and invariable pressure supply of CTs limited the practical applications and simultaneously caused uncomfortable feelings (Bar et al., 2021). Therefore, personalized CTs with individual-fitted morphology require pressure generation, and subjective EWCs need to be developed to improve functional therapeutic efficacy and user compliance.

In the biodesign and development processes of CTs, three-dimensional (3D) body scanning and anthropometric characterization are frequently utilized for body measurements with high accuracy and reasonable reliability (Kuzmichev et al., 2019; Yan et al., 2020). For instance, through the scanned 3D images and introduced non-uniform rational B-splines method, Wang et al. (2023) proposed a parametric modeling approach to efficiently reconstruct the lower limbs for customized design of CTs. However, in previous studies, the leg circumferences were characterized as the major referring design strategies of user-oriented CTs (Wang et al., 2023). The irregular cross-sectional shapes and varied curvatures of patient bodies frequently led to uneven and insufficient pressure generations (Li et al., 2022). The anatomic structural diversities of targeted users still need to be investigated to improve the clinic efficiency of CTs. Then, to facilitate the fabrication of CTs with custom fits, previous studies have modified Laplace’s law to predict the pressure magnitudes by calculating the material property variables, such as the circumferential mechanical tensions (Hui and Ng, 2001; Barhoumi et al., 2020), stretched ratios (Leung et al., 2010; Siddique et al., 2020), and material dimensional morphologies (Macintyre et al., 2004), etc. However, the corresponding inputting fabric’s physical–mechanical properties could only be achieved by performing multiple knitting attempts and excessive experimental tests. Furthermore, the parametric fitting equations relating to the yarn and machinery knitting variables were established to effectively achieve controllable fabric morphology and pressure behaviors of CTs (Shi et al., 2023; Wang et al., 2022). Nevertheless, the established equations were obtained through the sample measurements using leg mannequins with circular sectional profiles. The input interfacial pressure dosages in digital equations need to be optimized based on the irregular anatomic features of biological bodies. Thus, the pressure fitness and precise management of CTs need to be achieved through individual biological characterization, optimization of pressure values, and digitalization of designed knitting variables.

For estimations of personalized CTs, objective compression performances and subjective EWC assessments are conducted comprehensively to evaluate the medical therapeutic functions and patient adherences. First, instrumental pressure sensors and finite element (FE) modeling were applied for compression magnitude regional measurement and mapping visualization, respectively (Ghorbani et al., 2019; Ghorbani et al., 2020). The physical *in vivo* testing could directly estimate the specific generated pressure values at each leg position. Conversely, 3D FE models could visually provide the biomechanical distributions and profiles along lower limbs. Second, the EWCs of CTs were commonly evaluated through the movement ability and comfort sensation by actual wearing trails (Teyeme et al., 2021). Through various designed protocols, the postexperiment comfort survey (Baek et al., 2020; Cheng et al., 2022), visual analog scale (VAS) (Ke and Zheng, 2023), and wearing sensation questionnaire (Barhoumi et al., 2022) were performed for the EWC estimations and subjective preferences. Therefore, compression assessment systems of CTs could not only facilitate the evaluation of evidence-based treatment efficacy but also provide scientific information for material property optimization.

Consequently, to improve pressure fitness and EWCs for physical compression therapeutic modalities, personalized CTs are developed based on individual functional requirements and subjective comfortable perceptions. Through 3D reconstruction and body characterization, the geometric and morphologic variations of lower limbs were obtained as the biodesign strategies for the determinations of digitalized knitting variables. After 3D advanced seamless fabrication, the pressure performances and EWCs of CTs were evaluated by the proposed biomechanical FE CT-leg systems and VAS assessments, respectively. The present study will provide operable guidance for the design and manufacturing of biomedical therapeutic materials with precise pressure management and enhanced wearing comforts.

## Materials and methods

### Subject recruitment

**FIGURE 1:**
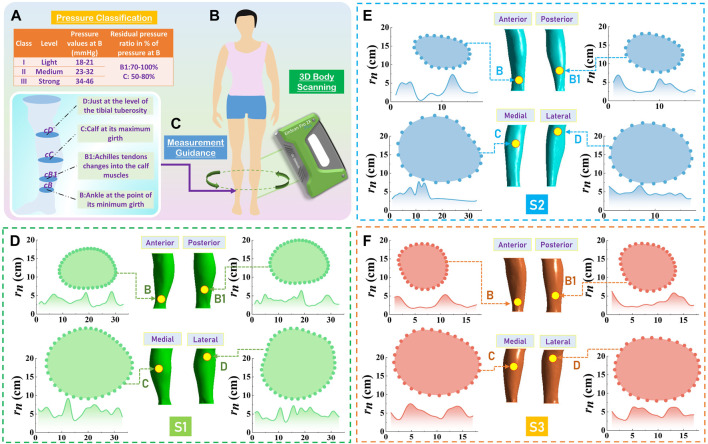
Three-dimensional (3D) body scanning and morphological characterization. **(A)** Pressure classification and gradient distribution through the Germany RAL-GZ 387 standard. **(B)** 3D body scanning for data acquisition using handheld EinScan-Pro 2X PLUS Scanner. **(C)** Clinic measurement guidance of lower limbs in compression therapy. The reconstructed lower extremity models, plotted biological curves, and the corresponding curvature radii of studied cross-sectional slices for subjects **(D)** S1, **(E)** S2, and **(F)** S3, respectively.

In the present study, three subjects (code: S1, S2, and S3) were voluntarily recruited at The Hong Kong Polytechnic University campus with various occupations and health conditions. Through the Clinical-Etiology-Anatomy Pathophysiology criteria and clinical presentation (Lurie and De, 2020), their prescribed pressure levels (S1-class I [18–21 mmHg]; S2-class II [23–32 mmHg]; and S3-class III [34–46 mmHg]) for physical-based compression therapy were referred to the Germany RAL-GZ 387 standard (Medical Compression Hosiery Quality Assurance; Figure 1A). The individual information and body mass index (BMI) of each subject were as follows: 1) S1: female, age: 51 years, height: 1.57 m, BMI: 25.6 kg/m^2^; 2) S2: male, age: 33 years, height: 1.80 m, BMI: 17.7 kg/m^2^; and 3) S3: female, age: 28 years, height: 1.68 m, BMI: 20.6 kg/m^2^.

### 3D reconstruction of lower extremity

To construct 3D lower limb models for body morphological characterization and biomechanical performance evaluation, the professional handheld EinScan-Pro 2X PLUS Scanner (Shining 3D Tech Co., Ltd. Hangzhou, China; scan precision: 0.4 mm; scan speed: 1.5 million dots/s) was applied for 2D image capturing (Figure 1B). During data acquisition process, participants were requested to stand steadily with torso separation and maintain a stable breathing state (Choi and Ashdown, 2011). Then, the initial Stereolithography (STL) files (right lower limbs) were imported into the Geomagic Studio 2014 (64 bit) software (Raindrop Geomagic, Research Triangle Park, NC, United States). The scanned derived cloud data was preliminarily processed for extra data elimination, noise reduction, and model hole repairment, respectively. Furthermore, the 3D entity leg models were reconstructed through reverse engineering technology using SpaceClaim Direct Modeler (SCDM; ANSYS, Pennsylvania, Pittsburgh, United States) and computer-aided design systems (Chockalingam et al., 2021). Then, the splattering points were obtained for further body characterization and data calculation.

### Anthropometric characterization and optimization of pressure distribution

**FIGURE 2:**
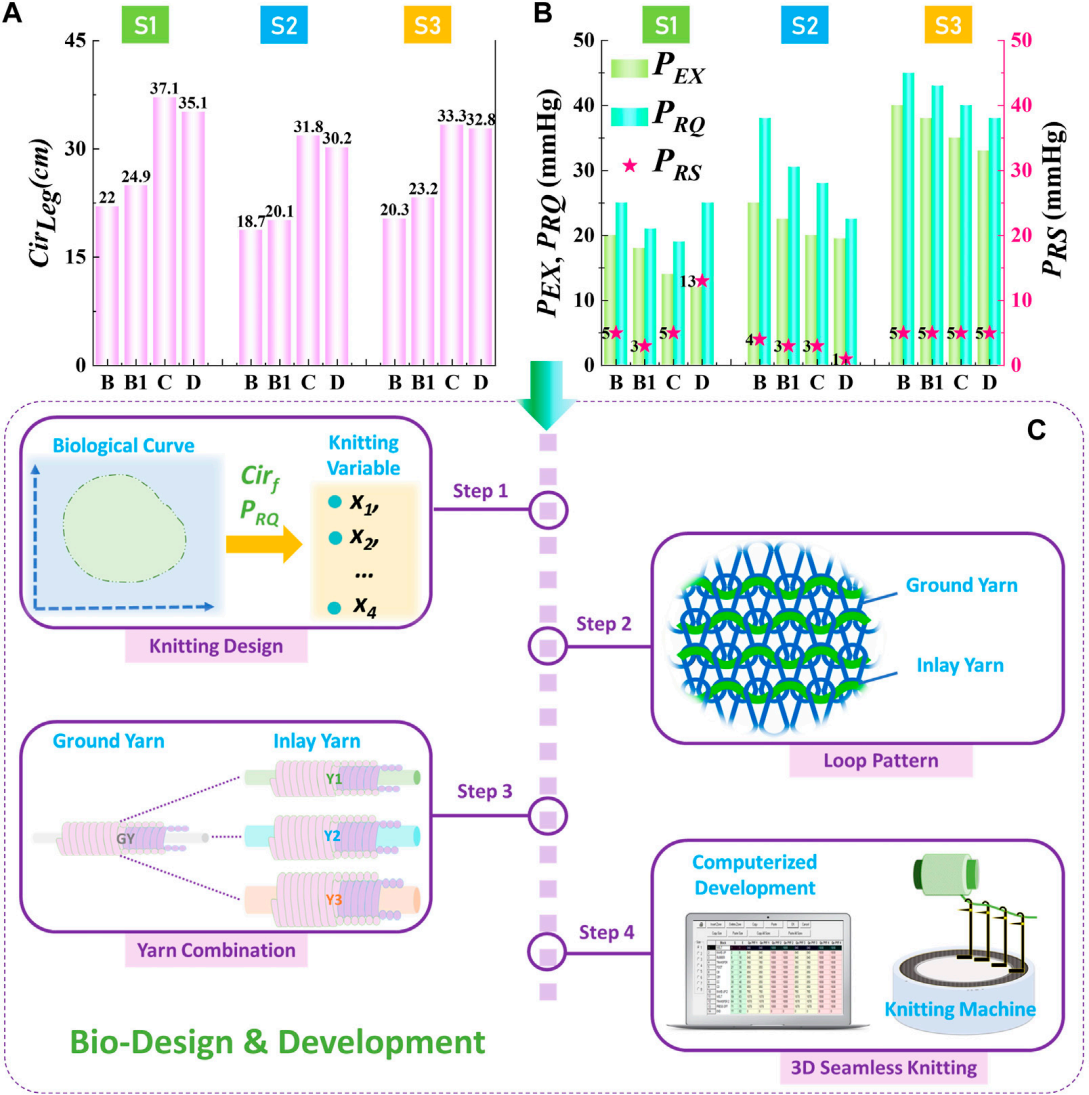
Pressure design and CT fabrication. **(A)** Leg dimensional circumferences were characterized as the basic size design strategy for the development of personalized CTs. **(B)** Practical pressure magnitudes of each leg position for each subject were optimized through their expected and reshaped pressure dosages. **(C)** Through body morphological profile characterization and compression performance optimization, the personalized CTs with 1 × 1 knitted structure were fabricated through the designed yarn combinations (ground yarn and inlay yarn) and machinery variables by the digital computerized development system and 3D seamless medical knitting machine.

Through the reconstructed lower extremity models, the digitalized 2D coordinate data was processed by using the MATLAB R2016a system (MathWorks Inc., Natick, MA, United States). According to randomized clinical experiments (Byrne, 2001; Prandoni et al., 2004), the below-knee length of compression stockings (CSs) with improved wearing comforts could efficiently prevent vein thrombosis and thrombotic syndrome. Thus, through the measurement guidance (Figure 1C), four (B, B1, C, and D) anatomic sites were determined as the major studied positions for the biofabrication of CTs. As shown in Figures 1D, E, the leg cross-sectional, biological curves were plotted by the sequential scattering points ([*x*_*i*_, *y*_*i*_], *i* = 1,2, …, *n*) for geometric calculation and shape recognition. Furthermore, the curvature radii (*r*_*1*_, *r*_*2*_,., *r*_*n*_) and curvatures ( $\frac{1}{r_{1}},\frac{1}{r_{2}},...,\frac{1}{r_{n}}$ ) of each slice were obtained by applying the Inline function, and dimensional circumferences (*Cir*_*Leg*_) of the subject’s lower limbs were calculated through $Ci{r_{L}}_{eg}=\sqrt{\sum_{i=1}^{n}{\left(x_{i+1}-x_{i}\right)}^{2}+{\left(y_{i+1}-y_{i}\right)}^{2}}$ (Figure 2A). In our previous study (Shi et al., 2024), the insufficient pressure supply caused by biological irregular shape diversities was optimized by the redistribution development guidance. In detail, based on the characterized standard deviations of curvature (*SDC*) values of each slice, the profile irregularities of leg cross-sections could be classified by our clustered irregular (IR) levels. Then, referring to the recommended optimization strategies, the reshaped pressure values (*P*_*R*S_) of each leg position for all subjects were obtained to scientifically enhance the delivered dosages of CTs. Through the standardized compression levels and gradient residual pressure ratios (Figure 1A), the specific expected pressure magnitudes (*P*_*EX*_) were designed, as illustrated in Figure 2B. Therefore, the required pressure values (*P*_*RQ*_; *P*_*RQ*_ = *P*_*EX*_ + *P*_*R*S_) were obtained as the functional individual requirements for further knitting variable design and achievements of precise compression management of CTs. For instance, for the B position of subject S1, the calculated *SDC* value and corresponding IR level were 1.25 and 2, respectively. Thus, through the reshaped *P*_*R*S_ of 5 mmHg and expected *P*_*EX*_ of 20 mmHg, the practical *P*_*RQ*_ dosage was optimized as 25 mmHg for further CS manufacturing.

### Biodesign and fabrication of personalized CTs

In compression development studies, synthetic yarns were commonly utilized to not only improve the elasticity and extensibility but also facilitate the strength and tenacity of knitted fabrics (Sathish et al., 2020). Therefore, the Nylon (Polyamide) double-covered Spandex (Polyurethane) yarn with linear density (D, Denier) of 40D/40D/40D (code: GY; yarn diameter: 0.27 mm) was technically used as the ground yarn, and the Nylon double-covered Spandex yarn with yarn count of 210D/40D/40D (code: Y1; yarn diameter: 0.41 mm), 280D/40D/40D (code: Y2; diameter: 0.42 mm), and 420D/40D/40D (code: Y3; yarn diameter: 0.47 mm) were adopted as the inlay yarns. Thus, in this study, three yarn combinations of GY/Y1, GY/Y2, and GY/Y3 were applied for the fabrication of personalized CTs with various material mechanical property requirements (Figure 2C). The circular seamless CTs were developed by the frequent-used 1 × 1 laid-in weft-knitted structure through a 3D LONATI LA-45 ME advanced medical knitting machine (Francesco Lonati, Brescia, Italy). Through existing experimental investigations (Machin et al., 2022), PYF feeding controller could adjust the feeding speed of inlay yarn materials and considerably influence the fabric circumferential morphologies. In addition, the sizing motor settings could control the loop lengths of knitted loops to vary fabric mechanical tensile behaviors (Shi et al., 2023). To effectively design the yarn and machinery variables, the quantitative relationships between the fabric circumferences (*Cir*_*f*_; cm) and pressure values (*P*_*RQ*_; mmHg) were fitted by our previous explorations through Eqs 1, 2, respectively (Shi et al., 2024). Thus, the specific applied yarn material combinations and machinery parameters for each customized CTs could be digitally determined. Moreover, the practical fabric course (circumferential) stretched ratios were referred to the Germany standard (from 15% to 120%), and inlay yarn diameter was used to replace the yarn linear density for simplifying the parametric calculations. Through the quantitative equations, the yarn-machinery knitting settings for each CTs were obtained as listed in Table 1.

$$Cir_{f}=0.016\times x_{1}+0.002\times x_{2}+84.493\times x_{3}-33.201,$$ (1)

$$P_{RQn}=-3.903-0.02\times x_{1}-0.062\times x_{2}+213.072\times x_{3}+0.217\times x_{4},$$ (2)

where *x*_*1*_ is the PYF feeding velocity of the inlay yarn (range: 550–1,300 m/min), *x*_*2*_ is the sizing motor setting values of knitted loops (range: 500–850 mm), *x*_*3*_ is the inlay yarn diameter (mm), and *x*_*4*_ is the fabric course tensile ratio (%) during the wearing state.

**TABLE 1:** Designed knitting settings of personalized CTs for recruited subjects

Applied user	Pressure level	Part	Yarn combinations	Inlay yarn diameter (mm)	*Cir*_*f*_ (cm)	PYF values (m/min)	Sizing motor values (mm)	Fabric stretch ratio (%)
S1	I	B	GY/Y1	0.41	17.60	1,010	700	25
		B1			19.92	1,150		
		C			22.22	1,300	850	67
		D			21.94	1,280	750	60
S2	II	B	GY/Y2	0.42	15.67	840	550	20
		B1			16.48	890	700	22
		C			20.38	1,130	750	56
		D			20.13	1,120	850	50
S3	III	B	GY/Y3	0.47	16.37	620	700	24
		B1			18.41	740		26
		C			20.68	890	850	61
		D			21.16	920		55

### Property investigations of CTs and determination of tissue stiffness

For the instrumental measurements of physical characteristics, fabric loop stitch densities (*LD*) were observed through clear microscopic images from Leica M165 C electronic device (Wetzlar, Germany) during the relaxed state in accordance with the ASTM D1577-79 standard test method. In addition, the fabric *Cir*_*f*_, thickness (*h*), and mass density (*MD*) of tubular CS knitted samples were tested based on the standards of ASTM D3774, ASTM D1777, and ASTM D 3776/D 3776M-09a, respectively. Additionally, the mean errors of *Cir*_*f*_ between the designed and tested values were calculated to evaluate the morphologic size fits of user-oriented customized CTs. For mechanical behaviors, the stress–strain curves, Young’s moduli (along the fabric course [*E*_*c*_] and wale [*E*_*w*_] directions) and Poisson’s ratios (*v*) were investigated by Instron 4411 uniaxial tension tester through ASTM D4964 standard. Shear moduli (*G*) of CTs were tested by the Kawabata (KES-FB3) pure shear testing system. To estimate the compression performance and pressure fitness of designed CTs, the practical interfacial pressure magnitudes (*P*) were measured at biological lower bodies by applying the Picopress^@^ pressure tester (Microlab Elettronica, Italy, precision: ±3 mmHg).

Through previous biological FE numerical models, the mechanical properties of soft tissue materials were frequently analyzed by Neo-Hookean model (Korhonen et al., 2011). In this study, the soft muscles—as the main compositions—were determined as the major studied tissue components of lower limbs (Ghorbani et al., 2020). Thus, the muscular tissues were assumed as the hyperelastic, isotropic, homogeneous, and incompressible materials. The constitutive functions are as follows (Chen et al., 2018):

$$W=C_{10}\left(\bar{I_{1}}-3\right)+D_{1}{\left(J-1\right)}^{2};C_{10}=\frac{S_{m}}{2};D_{1}=\frac{B_{m}}{2},$$ (3)

where *W* represents the strain energy density, $\bar{I_{1}}$ is the first strain variant, and *J* is the Jacobian determinant of the deformation gradient. *C*_*10*_ and *D*_*1*_ can be obtained under a linear elastic condition. *S*_*m*_ and *B*_*m*_ are the shear and bulk moduli, respectively. Additionally, for the incompressible materials, *J* value is 1.

Therefore, the subject individual tissue stiffness was measured using Aixplorer^@^ MultiWave ultrasound system (Supersonic Imagine, Aix-en-Provence, France). The tissue elasticities were physically tested using SuperLinear™ SL10-2 transducer array (element number: 192, bandwidth: 2–10 MHz). Then, the mechanical *C*_*10*_ values for subjects of S1, S2, and S3 (0.029 MPa, 0.020 MPa, and 0.031 MPa) were calculated by Eqs 4, 5.

$$E_{leg}=3\rho {v_{sw}}^{2},$$ (4)

$$S_{m}=\frac{E_{leg}}{2\left(1+v_{leg}\right)},$$ (5)

where *E*_*leg*_ and *ρ* are the tissue Young’s modulus and muscular density (1,000 kg/m^3^), *v*_*sw*_ is the shear wave velocity (0–7.7 m/s), and *v*_*leg*_ is the Poisson’s ratio (0.5; Dubois et al., 2018).

### Construction and validation of subject-specific biomechanical modeling

**FIGURE 3:**
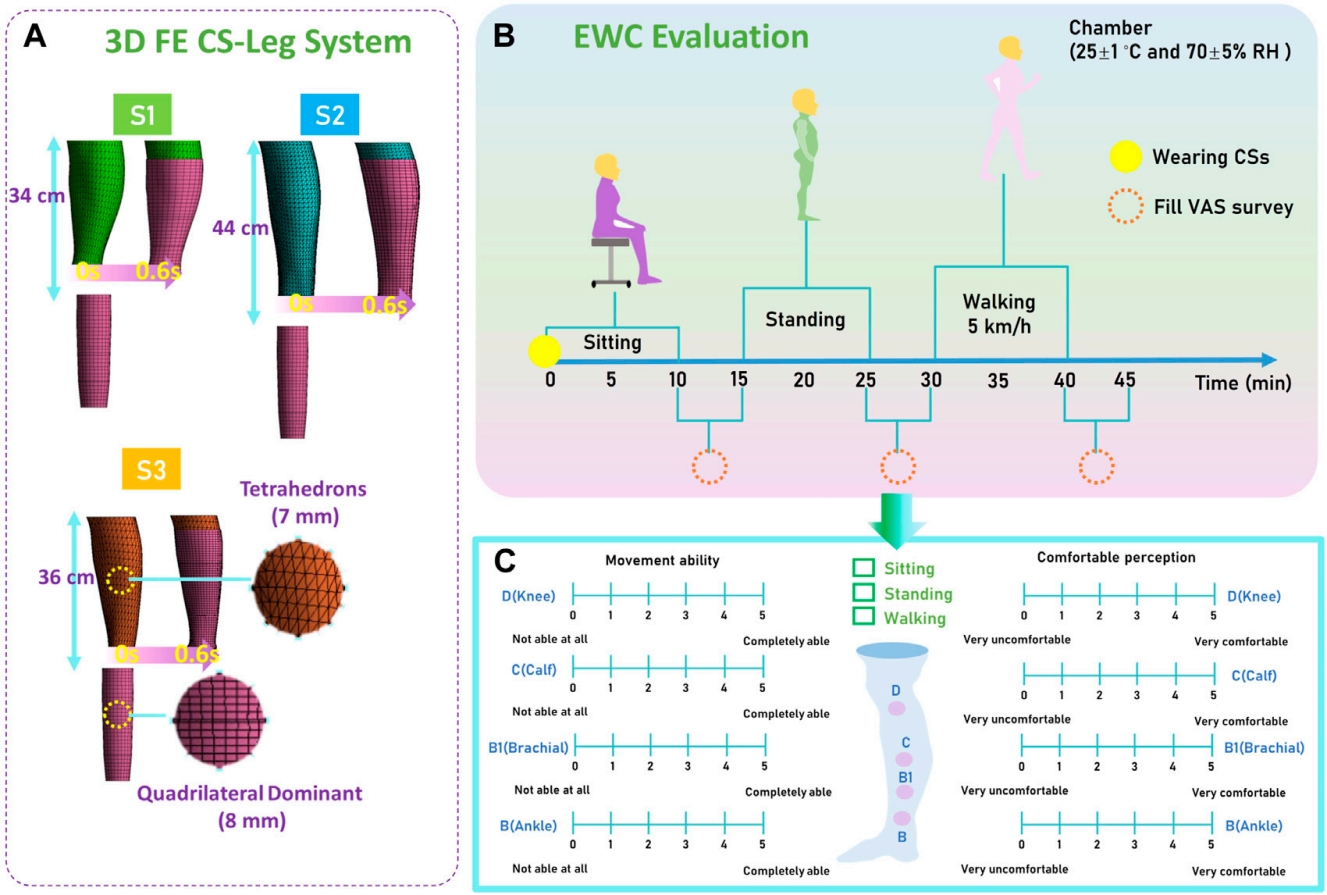
Numerical simulation and EWC evaluation protocol. **(A)** The established 3D FE CT-leg systems—including the geometric models, mesh sizes, mesh element types, and boundary conditions—for pressure performance visualization of recruited subjects of S1, S2, and S3. **(B)** Designed wearing trial experimental protocol for estimations of the movement ability and wearing comfort. **(C)** Utilized VAS scoring for EWC evaluation of developed user-oriented CTs.

3D FE CT-leg biomechanical modeling was established to visually evaluate the pressure performances of personalized CTs, as illustrated by Figure 3A. The geometric models of tubular CTs were constructed according to the actual designed fabric dimensions using ANSYS Workbench Design Modeler software (v19.2, ANSYS, Pennsylvania, Pittsburgh, United States). Then, the knitted CTs and lower limb models (established by Section *2*.*2*) were simultaneously imported into ANSYS LS-DYNA explicit solver to dynamically simulate the wearing process. Commonly, compression-knitted textiles were assumed as the orthotropic elastic materials in mechanical simulations (Weeger et al., 2018). Thus, the corresponding values of *MD*, *E*_*c*_, *E*_*w*_, *v*, and *G*, as well as the determined subject-specific tissue properties (*C*_*10*_), were imported into the CT-leg system as the inputting material characteristics. In addition, the CS (shell element; mesh size: 8 mm) and lower limb (solid entity element; mesh size: 7 mm) were meshed by Quadrilateral Dominant and Tetrahedrons elements, respectively. To simulate the sliding process, the upper and bottom leg surfaces were fixed to facilitate the freedom of CS sliding. In addition, the interfacial contact condition was applied to the frictional non-linear contact with a coefficient of 0.2 (Askari and Andersen, 2021). The longitudinal displacements (S1: 34 cm; S2: 44 cm; and S3: 36 cm) of CTs were determined as the boundary conditions of a biomechanical system for pressure behavior visualization. Moreover, to estimate the acceptability of proposed FE models, the validation study was conducted to compare the simulated and tested *p* values by *in vivo* measurements.

### Experimental protocol and data analysis for EWC evaluation

To evaluate the subjective EWC of designed customized CTs, VAS assessments were applied to estimate the movement ability and comfortable sensation by wearing trials. Participants were instructed to wear the personalized CTs in a controlled standard environment (temperature of 25°C ± 1°C and relative humidity of 70% ± 5%; ASTM D1776-04) for 24 h to achieve the equilibrium status prior to use. In detail, three phases were included to simulate the practical application scenarios (Leung et al., 2021; Figure 3B): 1) phase I: 10-min test in a sitting position for the stabilization of CTs and acclimatization; 2) phase II: 10-min static standing for active recovery; and 3) phase III: 10-min walk at a speed of 5 km/h as normal activity in daily life. Then, the critical VAS scoring forms with ratings of 1–5 were required to be filled by the participants during each phase interval (5 min for each survey fulfillment) (Figure 3C). Moreover, the Pearson correlation tests were conducted to further investigate the relationship between the compression levels with the CT EWCs (Manfei et al., 2017). The collected ranking scores for EWC evaluation were analyzed by Statistical Package for the Social Sciences software (version 23.0, IBM Corporation, United States). Additionally, the level of significance was set at α = 0.05 (Amrherin et al., 2017).

## Results and discussion

### Physical–mechanical properties and morphologic fits of developed personalized CTs

**FIGURE 4:**
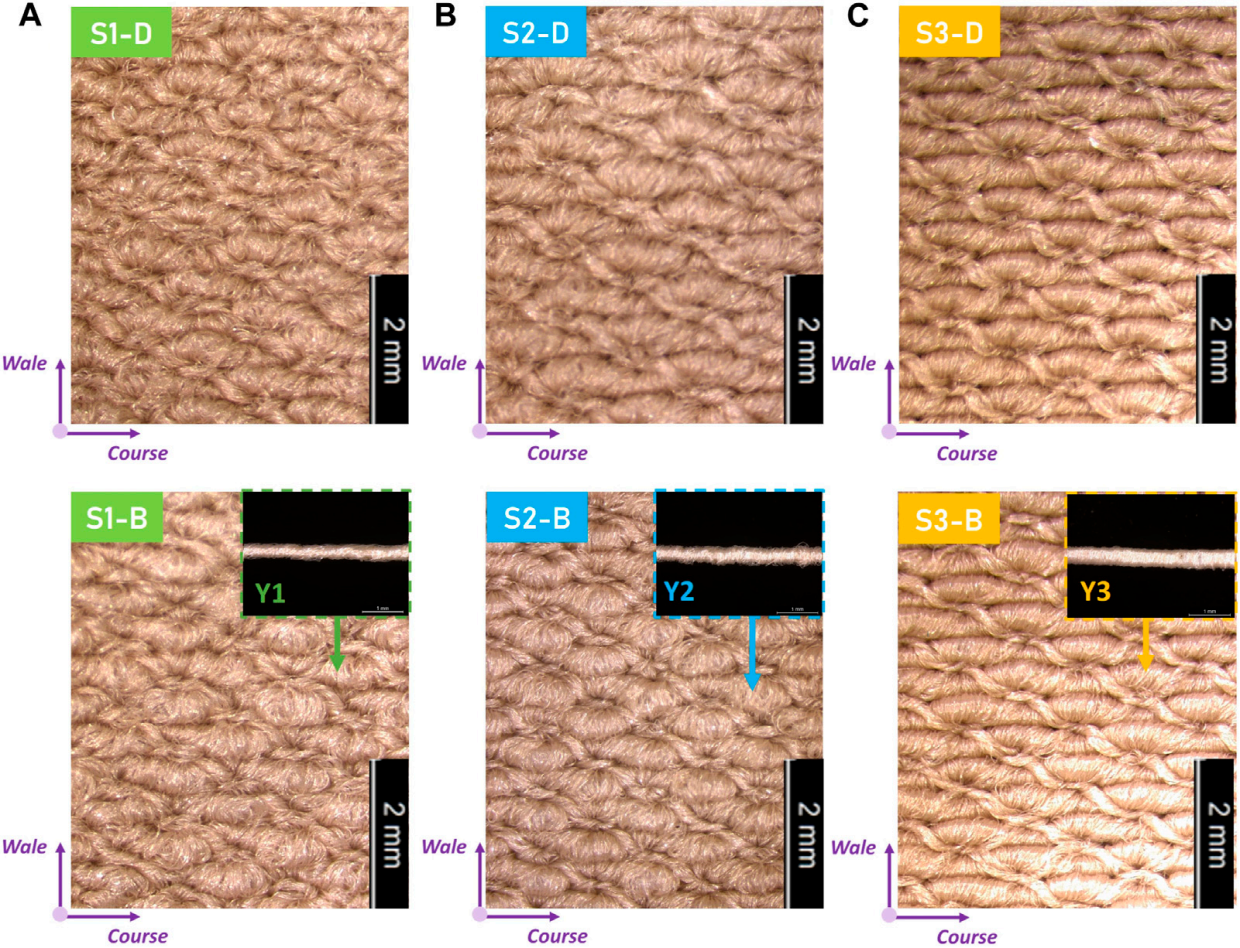
Microscopic investigations of designed CTs. Images of developed knitted CS samples for subjects **(A)** S1, **(B)** S2, and **(C)** S3, respectively.

**FIGURE 5:**
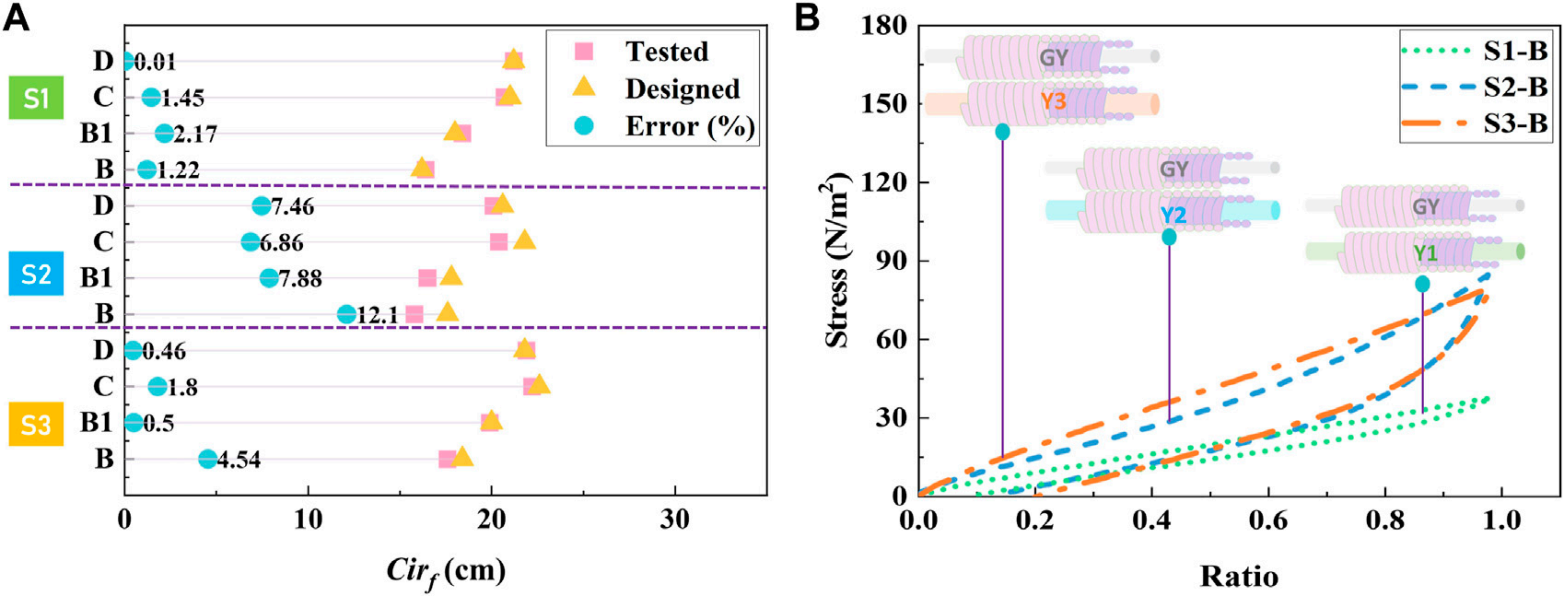
Fabric circumferential characteristics and tensile properties. **(A)** Fabric morphology comparisons between the designed and fabricated personalized CTs. **(B)** Mechanical tensile behaviors of knitted CS samples (stocking part of B) with various yarn combinations along the fabric course (circumferential) stretching direction.

Through the experimental sample testing, the physical–mechanical properties of each studied stocking part of developed personalized CTs were listed in Table 2. First, accepted for fabric *h*, the physicla *LD* and *MD* of knitted CTs varied related to the applied yarn combinations and machinery parameters (Liu et al., 2013). Generally, fabric *LD* was decreased from the B to D stocking parts varied by the formed loop sizes through the adjustment of loop length settings (Figure 4). Moreover, for different compression levels of CS shells, the increased inlay yarn diameters positively influenced the fabric *MD*. Second, as shown in Figure 5A, the mean error ratio of fabric *Cir*_*f*_ between the designed and fabricated CTs was approximately 3.87% ± 3.70%. Thus, the stocking dimensions could achieve the basic size fitness for each subject (Wang et al., 2022). Third, for the mechanical tensile behaviors, through the physical mechanisms of Laplace’s law, the generated compression magnitudes were fundamentally determined by the textile tensions and Young’s moduli along the fabric circumferential stretched directions (Shi et al., 2024). Therefore, the measured *E*_*c*_ values varied for material mechanical requirements caused by the designed loop length settings and applied yarn combinations. In detail, as illustrated in Figure 5B, the knitted samples (yarn combination: GY/Y3) for compression of class III could generate high material tensions under the identical uniaxial stretched ratios. Thus, the applied inlay yarn thickness could positively impact the material mechanical stiffness for the required pressure generations.

**TABLE 2:** Measured fabric physical–mechanical properties of fabricated CTs

Applied user	Part	Physical properties			Mechanical properties			
		*h* (mm)	*LD* (stitches/cm^2^)	*MD* (g/m^2^)	*E*_*c*_ (MPa)	*E*_*w*_ (MPa)	*v*	*G* (MPa)
S1	B	0.63	426.72	481.27	0.38	0.21	0.20	0.16
	B1	0.63	426.72	481.13	0.37	0.21	0.20	0.16
	C	0.65	416.37	468.19	0.33	0.15	0.21	0.14
	D	0.63	406.33	481.27	0.37	0.19	0.20	0.15
S2	B	0.62	414.03	510.20	0.57	0.34	0.20	0.24
	B1	0.62	349.98	529.95	0.43	0.23	0.22	0.18
	C	0.62	345.16	503.62	0.42	0.18	0.22	0.17
	D	0.64	341.64	481.51	0.41	0.17	0.23	0.17
S3	B	0.64	305.74	532.74	0.76	0.27	0.25	0.30
	B1	0.64	302.58	532.55	0.75	0.27	0.25	0.30
	C	0.65	302.10	521.61	0.61	0.19	0.27	0.24
	D	0.65	300.48	521.51	0.60	0.19	0.27	0.26

During the wearing process, stretched CTs were provided sustainable tensions for interfacial pressure generations (Fun et al., 2011). The corresponding fabric tensile ratios were determined by the dimensional diversities between CTs and applied biological bodies. For ready-made commercial CTs, the fabric *Cir*_*f*_ of each part was designed by various manufacturers for extensive user groups. Nevertheless, the provided size tables and recommended selections could not simultaneously conform to the leg morphological variables. The oversized CTs could not generate the required pressure dosages along the low extremities. Conversely, the undersized CTs may lead to excessive compressions, unexpected tourniquet effects, and wearing discomforts (Lozo et al., 2021). Thus, in this study, based on the 3D-body scanning and reverse engineering technologies, biological geometric circumferences and morphologic profiles of each subject were characterized by the obtained anthropometric data. Through the calculated *Cir*_*Leg*_ values and standardized stretch ratios (15%–120%), *Cir*_*f*_ of each part for user-oriented CTs was achieved through designed yarn-machinery knitting settings. Therefore, the customized CTs based on individual leg geometric parameters could facilitate the achievement of pressure precision and patient compliance (Yang et al., 2023).

### Validation of proposed subject-specific FE CT-leg system

**FIGURE 6:**
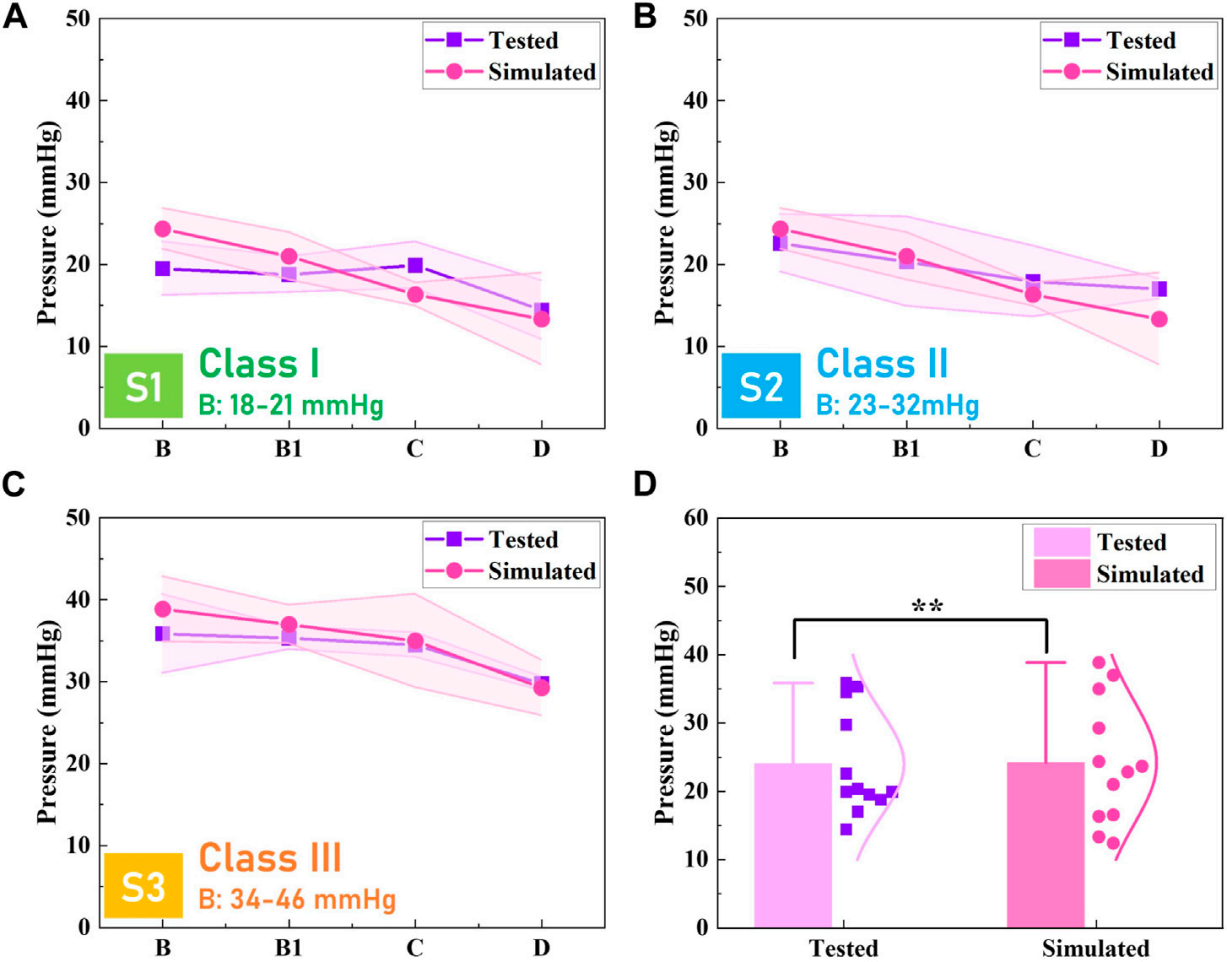
Validation results of FE systems. Experimental measurement results and simulated data by FE CT-leg systems for subjects **(A)** S1, **(B)** S2, and **(C)** S3, respectively. **(D)** Pressure comparisons and data correlation between the tested and simulated values of prepared customized CTs.

To validate the prediction precision and acceptability of the proposed subject-specific FE models, the measured and simulated pressure values of each CT were compared, as shown in Figure 6. For example, the tested pressure magnitudes at the leg positions of B, B1, C, and D for subject S1 were approximately 19.49 ± 3.27 mmHg, 18.75 ± 2.18 mmHg, 19.91 ± 2.85 mmHg, and 14.42 ± 3.59 mmHg, respectively (Figure 6A). Similarly, the simulated pressures were approximately 22.84 ± 5.41 mmHg, 23.67 ± 6.42 mmHg, 16.55 ± 7.71 mmHg, and 12.41 ± 4.48 mmHg, respectively. For the customized CTs prepared for subject S3, the prediction error ratios for studied lower limb positions of B, B1, C, and D were 8.43%, 4.73%, 1.42%, and 1.68%, respectively (Figure 6C). Through calculations, the averaged error ratio of established FE CT-leg modeling was approximately 11.00% ± 7.78%. Furthermore, Pearson test results showed that the measured results had significant correlations (Sig. <0.05, *ρ* = 0.96) with the simulated data (Figure 6D). Thus, the constructed FE mechanical modeling with subject morphologic characteristics and biomechanical properties could be accurately utilized for the pressure prediction and mapping visualization of CTs.

Previously, pressure evaluations were commonly performed by instrumental sensor devices through *in vivo* testing. However, for biological bodies—due to the individual anatomic structural characteristics—the irregular interfacial body surfaces and sectional curvatures could lead to uneven and insufficient pressure generations. Thus, the obtained regional magnitudes could not reflect the practical pressure profiles distributed by applied CTs. Moreover, in relevant compression simulation models—the pressure performances—biomechanical transmission behaviors of internal tissue stress and venous hemodynamics could be simulated by constructed 3D modeling. Nevertheless, their geometric body models were established by excessive scanned slices through magnetic resonance imaging or computed tomography scanning (CT). For our proposed FE system, through the 3D-body scanning and reconstructed lower extremity models, the visualization of pressure performances could be efficiently achieved. Additionally, the predicted errors of existing models ranged from 6.0% to 21% (Ghorbani et al., 2019; Ghorbani et al., 2021). Therefore, the proposed FE CT-leg system could be applied for pressure performance evaluation and material property optimization with high prediction accuracy and simulation efficiency.

### Pressure performance evaluation of developed personalized CTs

**FIGURE 7:**
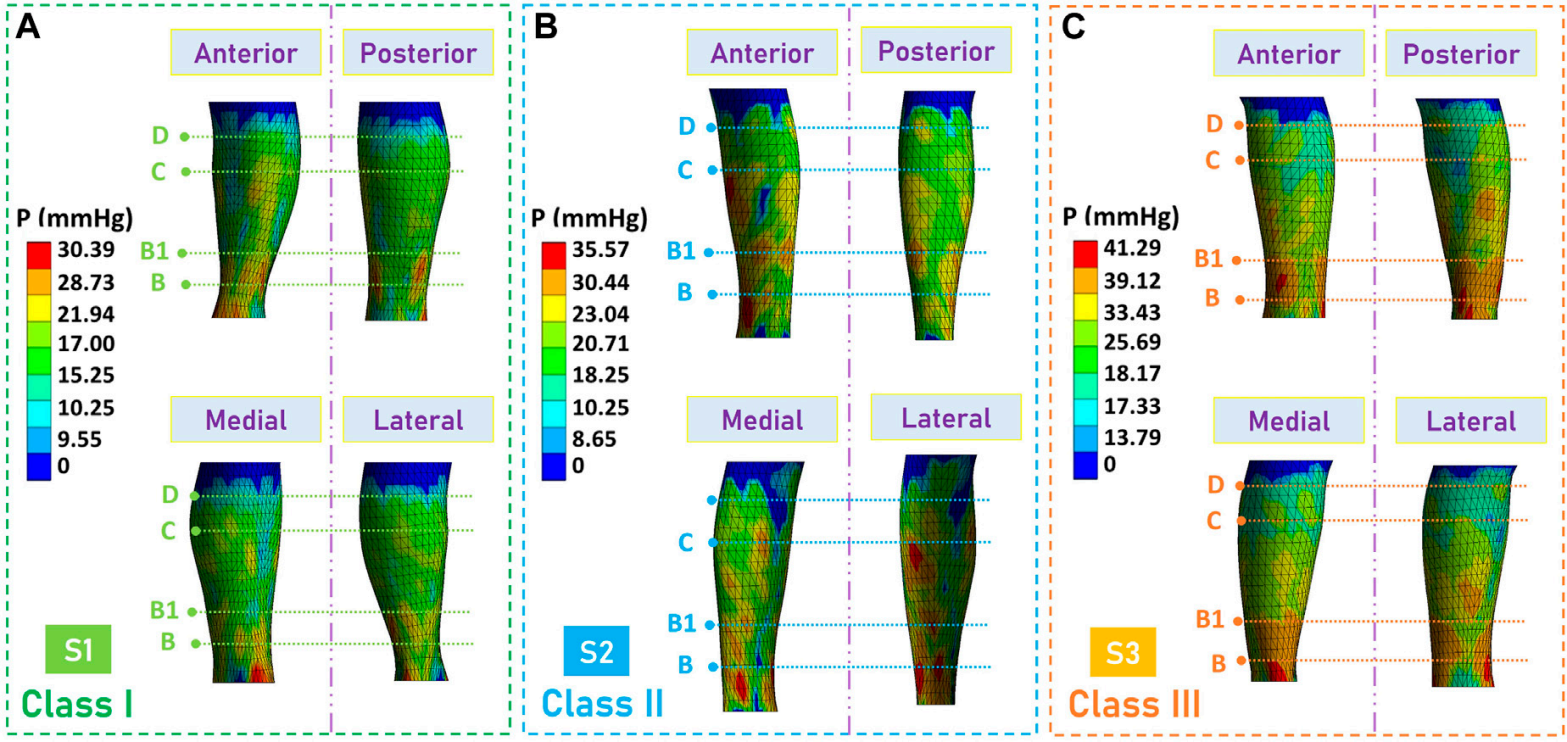
FE simulated compression results. Pressure profile mappings obtained by FE CT-leg modeling systems for subjects **(A)** S1, **(B)** S2, and (3) S3, respectively.

**FIGURE 8:**
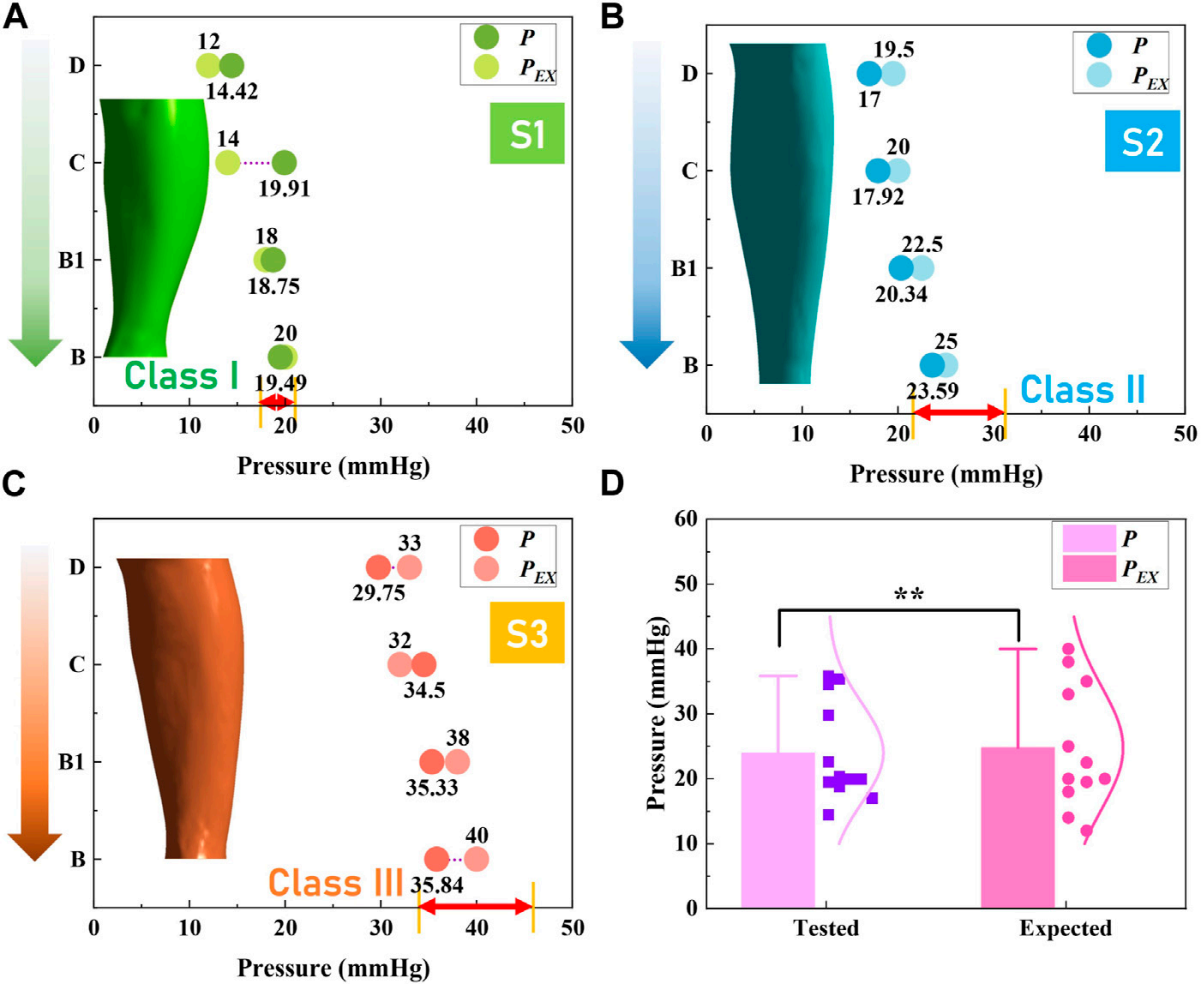
Pressure performance evaluation results. Pressure distribution evaluations of the developed customized CTs for subjects **(A)** S1, **(B)** S2, and (3) S3, respectively. **(D)** Data comparison between the user expected and actual tested interfacial pressure dosages of CTs.

Through the proposed 3D FE modeling, the pressure mappings exerted by developed CTs along lower extremities of each subject were visualized, as shown in Figures 7A–C. Generally, the gradient pressure distributions were regressive from the distal to the proximal regions generated by customized CTs through the development processes. Furthermore, to evaluate the pressure fitness of CTs, the expected compression exertions (*P*_*EX*_) and practical pressure values (*P*) were compared, as shown in Figure 8. In detail, referring to the Germany standard (Figure 1A), subject S1 expected compressions (class I) ranged from 18 mmHg to 21 mmHg and the tested pressure dosage at the leg B position was 19.49 ± 3.27 mmHg (Figure 8A). For subjects S2 (Figure 8B) and S3 (Figure 8C), the delivered interfacial pressures were 23.59 ± 3.51 mmHg and 35.84 ± 4.80 mmHg, respectively. Moreover, through correlation analysis, the tested *P* data had good agreements (Sig. <0.05, *ρ* = 0.97) with the designed *P*_*EX*_ values (Figure 8D), and the mean pressure error ratio was 10.08% ± 7.75%. Therefore, the developed personalized CTs could achieve the pressure fitness and medical therapeutic functions for applied users with various requirements.

Moreover, in previous studies (Gao et al., 2015), customized CTs were developed according to the measured *Cir*_*Leg*_ values and expected pressure levels. The pressure error ratios between the designed and practical tested values were approximately 5%–52% (Wang et al., 2023). However, in development process, their referred quantitative relationships between the yarn-machinery variable with pressure values were fitted through the leg mannequin measurement with ideal circular cross-sectional profiles. Thus, in the present study, to promote pressure fitness, the insufficient compression exertions caused by individual irregular leg shapes were scientifically improved based on the reshaped biodesign guidance. In addition, through the performance evaluated results, the compression levels and gradient distributions of each developed CS conform to the Germany standard and specific pressure prescription. Thus, the proposed biodesign and development strategies could be utilized for manufacturing CTs with extensive personal requirements in healthcare, medical, and rehabilitation domains (Aydin et al., 2019).

### EWC evaluation of developed personalized CTs

**FIGURE 9:**
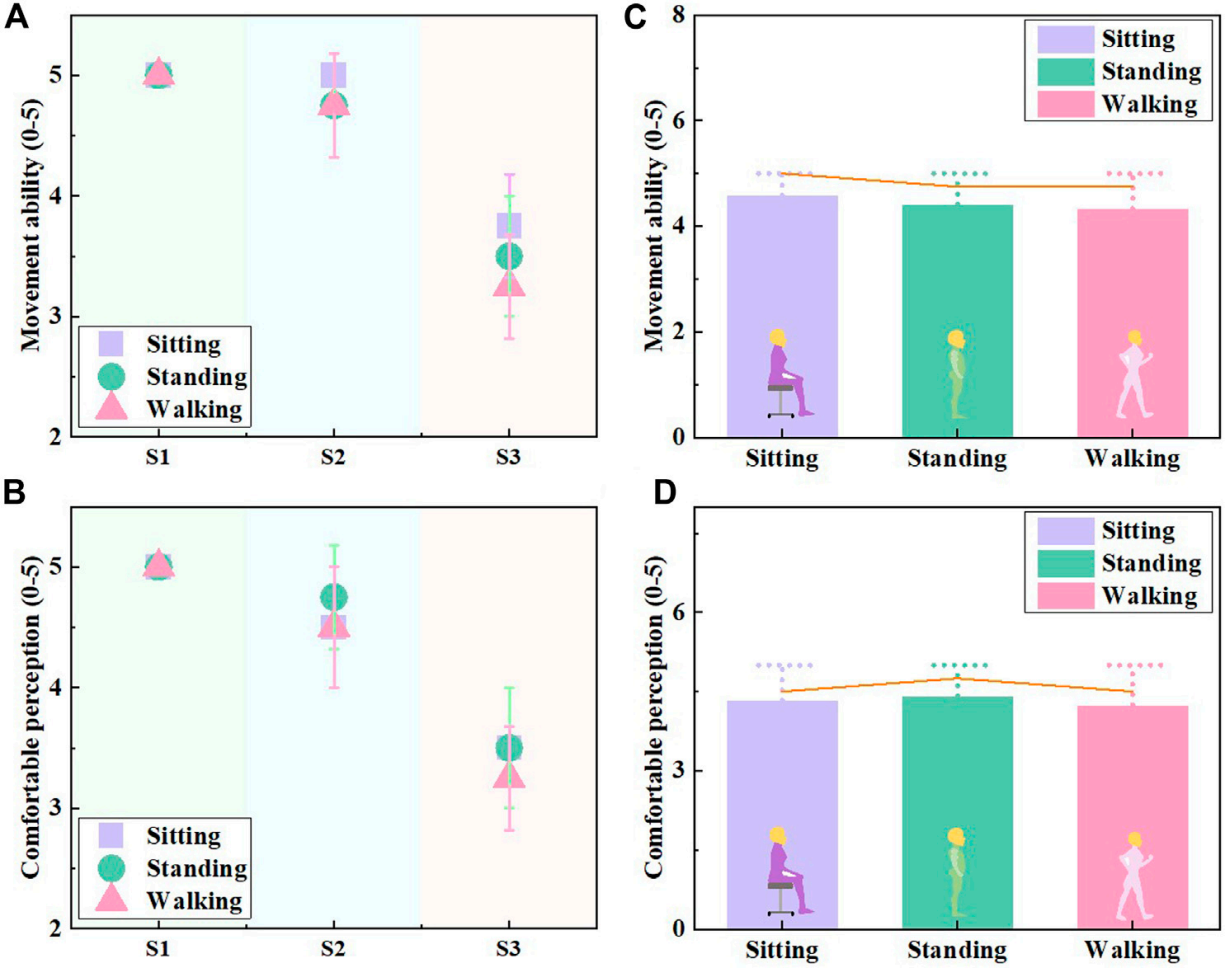
Subjective assessment results of EWC. **(A)** Movement ability and **(B)** comfortable perception assessments of the developed customized CTs for EWC evaluations. **(C)** Movement ability and **(D)** comfortable perception comparisons during various applied body postures.

The subjective VAS scoring results through the subject wearing trials for EWC assessments of fabricated CTs are shown in Figure 9. Generally, the developed customized CTs for each subject had acceptable movement abilities and wearing comforts in practical application (Kırcı et al., 2021). The elastic knitted CTs for subject S1 had flexible mobility and comfortable perception during the sitting, standing, and walking states because of relatively lower stocking compression of class I (Figures 9A, C). Thus, the fabric stretched tensions for lower levels of pressure generation positively enhanced EWCs of therapeutic compression biomaterials. Through correlation analysis, the generated increasing compression classes significantly decreased (Sig. <0.05, *ρ* = −0.92) the CT EWCs. Furthermore, through applied scenario comparisons, during the dynamic processes of muscular activity, fabric mechanical tensile stresses varied by leg morphological variations led to an uncomfortable feeling and moveable limitation. Therefore, compared with the active walking states, the sitting and standing postures could subjectively benefit the movement ability and wearing comfort, respectively (Figures 9B, D).

The material design, structural development, and fabrication technique crucially contributed to the CT EWCs. In the present study, the selections of double-covered yarn materials improved the fabric flexibility based on its excellent mechanical elastic recovery properties. For applied yarn materials, the increased yarn diameters provided more fabric stress during the stretching process. Thus, CTs with yarn combinations of GY/Y3 generated high pressure performances along the low limbs. Sequentially, the larger fabric tensions had negative impacts on practical CT EWC. Moreover, for the designed 1 × 1 laid-in loop pattern, the combinations of the ground and inlay yarn components could provide the basic fabric dimensional stabilities during the wearing stretching process. For the machinery parameter design, the stocking dimensional profiles and gradient mechanical material stiffness were achieved by varying the inlay yarn feeding speeds and loop lengths. Therefore, the proper material morphology, functional pressure distribution requirements, and allowing freedom of body movement simultaneously promoted EWCs in practical application. In our future work, natural and biodegradable fibers will be utilized as the yarn compositions to enhance the comfortable perceptions and moisture management for improving wearing comforts.

Furthermore, for prescribing the clinical efficacy of compression therapy, the static stiffness index of CTs reflects the ability to counter the muscle expansion during contraction. Conversely, the dynamic stiffness index indicates the pressure pulsation capability in dynamic wear (Partsch, 2005; Partsch et al., 2006). Through previous studies (Liu et al., 2020; Wang et al., 2023) on the development processes, only the morphological characteristics of leg circumferences were measured as CT design strategies. The individual curvature variations frequently led to inaccuracy or insufficient pressure generations of fabricated CTs. For the evaluation of CTs, only regional interfacial pressure magnitudes along the lower extremities were tested through instrumental sensor devices, lacking pressure mapping profiles and practical wearing perceptions. Thus, the practical functional performances of designed custom fit CTs would be estimated during various human activities in further study.

## Conclusion

The present study fabricated the personalized therapeutic CTs for the needed users with individual morphologic and compression requirements. Through reverse engineering technology and body anthropometric characterization, the anatomic biological profiles and geometric variables were obtained as the basic design strategies of material morphology and pressure optimization. The applied knitting yarn combinations and machinery parameters were digitally obtained for the 3D seamless fabrication. Then, through the reconstructed geometric lower extremity models and determined tissue stiffness, the subject-specific 3D FE CT-leg modelings with high prediction accuracy (error ratio: 11.00% ± 7.78%) were established for the biomechanical evaluation of pressure performances. Based on the practical wearing trials, the interfacial compressions of developed customized CTs had good agreements with the expected data (pressure error ratio: 10.08% ± 7.75%). Moreover, through subjective VAS assessments, the CT EWCs had significant (Sig. <0.05, *ρ* = −0.92) correlations with the exerted pressure levels. Thus, through the proposed digital design, development, and evaluation systems, this study provided a comprehensive manufacturing approach for the therapeutic biomedical materials and promotes the precise pressure management for textile-based compression therapy.

## Data Availability

The original contributions presented in the study are included in the article/supplementary material. Further inquiries can be directed to the corresponding author.
